# Temporomandibular Disorders as a Risk Factor for Suicidal Behavior: A Systematic Review

**DOI:** 10.3390/jpm12111782

**Published:** 2022-10-28

**Authors:** Vittorio Dibello, Francesco Panza, Giorgio Mori, Andrea Ballini, Michele Di Cosola, Madia Lozupone, Antonio Dibello, Filippo Santarcangelo, Vincenzo Vertucci, Mario Dioguardi, Stefania Cantore

**Affiliations:** 1 Department of Orofacial Pain and Dysfunction, Academic Centre for Dentistry Amsterdam (ACTA), University of Amsterdam and Vrije Universiteit Amsterdam, 1081 Amsterdam, The Netherlands; 2 Unit of Research Methodology and Data Sciences for Population Health, National Institute of Gastroenterology and Research Hospital IRCCS “S. De Bellis”, Castellana Grotte, 70013 Bari, Italy; 3 Department of Clinical and Experimental Medicine, University of Foggia, Via Rovelli 50, 71122 Foggia, Italy; 4 Department of Precision Medicine, University of Campania “Luigi Vanvitelli”, 80138 Naples, Italy; 5 Neurodegenerative Disease Unit, Department of Basic Medicine, Neuroscience, and Sense Organs, University of Bari Aldo Moro, 70124 Bari, Italy; 6 Accident and Emergency Department (AED), Fabio Perinei Hospital, Altamura, 70022 Bari, Italy; 7 Private Practice, 70100 Bari, Italy; 8 Private Practice, 88900 Crotone, Italy; 9 Independent Researcher, Regional Dental Community Service “Sorriso & Benessere-Ricerca e Clinica”, 70129 Bari, Italy

**Keywords:** orofacial pain, temporomandibular disorders, temporomandibular joint dysfunction, suicidal behavior, suicidal ideation, suicide attempts, suicide completion, chronic pain

## Abstract

Background: Temporomandibular disorders (TMD) are a group of common musculoskeletal dysfunctions that affect the temporomandibular joint or masticatory muscles and related structures or are expressed as a clinical combination of these two factors. The etiology of TMD is multifactorial and features related to anxiety, depression and mental disorders can contribute to the predisposition, onset and progression of TMD. The ability to adapt and develop coping attitudes was reduced in patients presenting with chronic pain, while suicidal behavior (suicidal ideation, suicide attempts, and suicide completion) was increased. The objective of this review was therefore to investigate suicidal behavior in relation to TMD. Methods: The review was performed according to the PRISMA 2020 guidelines. Six databases (PubMed, MEDLINE, EMBASE, Scopus, Ovid, and Google Scholar) were consulted through the use of keywords related to the review topic. The study is registered on PROSPERO (CRD42022320828). Results: The preliminary systematic search of the literature yielded 267 records. Excluding duplicates, 15 were considered potentially relevant and kept for title and abstract analysis. Only six articles were considered admissible reporting a single exposure factor, TMD and a single outcome, suicidal behavior, although these were evaluated through different assessment tools. We found a low association of TMD with suicidal behavior in observational studies, with estimates partly provided [prevalence ratio (PR) from 1.26 to 1.35, 95% confidence intervals (CI) from 1.15 to 1.19 (lower) and from 1.37 to 1.54 (higher); and odds ratios (OR) from 1.54 to 2.56, 95% CI from 1.014 to 1.157 (lower) and 2.051 to 6.484 (higher)], a relevant sample size (*n* = 44,645), but a few studies included (*n* = 6). Conclusions: The results of the included studies showed that the prevalence data of suicidal behavior were more present in young adults with TMD, with a controversial association with gender. Suicidal behavior was also correlated and aggravated by the intensity of pain.

## 1. Introduction

Temporomandibular disorders (TMD) are a group of common musculoskeletal dysfunctions that affect the temporomandibular joint (TMJ) or masticatory muscles and related structures or are expressed as a clinical combination of these two factors. Constituting the main cause of oro-facial pain of non-odontogenic origin, they recognize a multifactorial etiology, and many aspects of the onset and progression are still to be clarified or defined [1].

The symptom presentation ranges from the presence of noises related to the TMJ, to muscle pain in the face and in the areas adjacent to the ear, often with pain on palpation up to difficulty opening the mouth with the concomitant presence of pain. Symptoms may occur spontaneously in the absence of stimuli or may present with exacerbation in relation to the chewing, speech and swallowing function or in the presence of a yawn [2].

The incidence was related to age and the prevalence data reported that 10% of the adult population was affected, the prevalence tended to be low in children with a peak in the young adult and a decline after the 5th decade of life [3].

The etiology of TMD is multifactorial and biomechanical, neuromuscular, biopsychosocial, and biological factors may contribute to these disorders [4,5,6,7]. In particular, aspects related to anxiety, stress, depression [8], and more generally, mental/psychological disorders can contribute to the predisposition, onset and progression of this disorder [9]. The ability to adapt and develop coping attitudes were reduced in patients presenting with chronic pain and with depression and anxiety, and suicidal behavior (suicidal ideation, suicide attempts, and suicide completion) was increased in these patients [10,11].

Prevalence data in subjects with chronic pain of suicidal ideation, suicide attempts and suicide completion ranges from 7 to 40% [12,13,14,15,16]. TMD is the third most prevalent chronic painful disease, following tension headaches and back pain [14]. A recent study by Park and colleagues (2020) on data from the fifth KNHANES (Korean National Health and Nutrition Examination Survey), that includes a cohort of 25,533, of which only 8049 met the inclusion criteria, reported how suicidal ideation was related to TMD, especially in female subjects [16]. However, the scientific literature was not very extensive, and prevalence data on suicidal behavior in the population suffering from TMDs were few and lacked a single method of assessment [17]. To the best of our knowledge, the present study is the first systematic review investigating these two conditions. The aim of the study was to investigate and to report all prevalence data and diagnostic tools used in the included observational studies.

## 2. Materials and Methods

### 2.1. Search Strategy and Data Extraction

The present systematic review followed the Preferred Reporting Items for Systematic reviews and Meta-Analyses (PRISMA) 2020 guidelines, adhering to the PRISMA 2020 27-item checklist [18]. We performed separate searches in the US National Library of Medicine (PubMed), Medical Literature Analysis and Retrieval System Online (MEDLINE), EMBASE, Scopus, Ovid, and Google Scholar databases to find original articles enquiring about any association between TMD (exposure) and suicidal behavior (outcome). The exposure factors were selected to include any indicator(s) of TMD, regardless of the measurement method (clinical examination or self-reported evaluation), while the outcome(s), included any content assimilated to suicidal behavior such as suicidal ideation, suicidal thoughts, self-harm, self-directed violence, attempted and completed suicide, and parasuicide.

The search strategy used in PubMed and MEDLINE, and adapted to the other four electronic sources, is shown in Supplementary Table S1. The literature search covers the timeframe from the database inception to March 25th, 2022. No language limitation was introduced. Two investigators (V.D., A.B.) searched for papers, screened titles, and abstracts of the retrieved articles separately and in duplicate, checked the complete texts, and selected records for inclusion. In addition, a third reviewer (M.D.) was tasked with resolving doubtful situations.

### 2.2. Protocol and Registration

An *a priori* protocol was established and registered, without particular amendments, to the information provided at registration, on PROSPERO (Centre for Reviews and Dissemination, University of York; http://www.crd.york.ac.uk/PROSPERO), a prospective international register of systematic reviews (registration number—CRD42022320828, in date 25 April 2022). No skimming was applied to the recruitment settings (home care, hospital, community) or general health status and the age of the involved subjects. Technical reports, letters to the editor, and systematic and narrative review articles were excluded.

The following information was extracted by the two investigators (V.D., S.C.), separately and in duplicate, in a piloted form: (1) general information of single studies (author, year of publication, country, settings, design, sample size, age); (2) exposure assessment methods (World Health Organization (WHO) criteria; research diagnostic criteria for TMD (RDC/TMD); interviews and oral examinations); and (3) outcome assessment tools (single or multiple questions from validated and non-validated questionnaires).

All references selected for retrieval from the databases were managed with the commonly used MS Excel software platform for data collection. Afterward, all duplicated records were excluded. Potentially eligible articles were identified by reading the abstract and, if necessary, by reading the full-text version of the articles. Data were cross-checked, any discrepancies were discussed, and disagreements were resolved by a third investigator (M.D.). Lastly, data extracted from selected studies were structured in tables of evidence.

### 2.3. Quality Assessment within and across Studies and Overall Quality Assessment

The methodological quality of included observational studies was independently appraised by paired investigators (V.D. and M.D.), using the National Institutes of Health Quality Assessment Toolkits for Observational Cohort and Cross-Sectional Studies [19]. Ratings of high (good), moderate (fair), or poor were assigned to studies according to the criteria stated in the toolkit. This tool contains 14 questions that assess several aspects associated with the risk of bias, type I and type II errors, transparency, and confounding factors, i.e., study question, population, participation rate, inclusion criteria, sample size justification, time of measurement of exposure/outcomes, time frame, levels of the exposure, defined exposure, blinded assessors, repeated exposure, defined outcomes, loss to follow-up, and confounding factors. Items 6, 7, and 13 do not refer to cross-sectional studies and the maximum possible scores for cross-sectional and prospective studies were 8 and 14, respectively. Disagreements regarding the methodological quality of the included studies were resolved through discussion until a consensus was reached or resolved by a fourth investigator (F.P.). The Grading of Recommendations Assessment, Development and Evaluation (GRADE) rating system was used to assess the overall certainty of evidence of the studies included in the present systematic review [20]. The following factors were considered across the evidence base: risk of bias; inconsistency of results; indirectness of evidence; imprecision; and publication bias. GRADE certainty of evidence was graded as high, moderate, low, and very low.

## 3. Results

**Figure 1:**
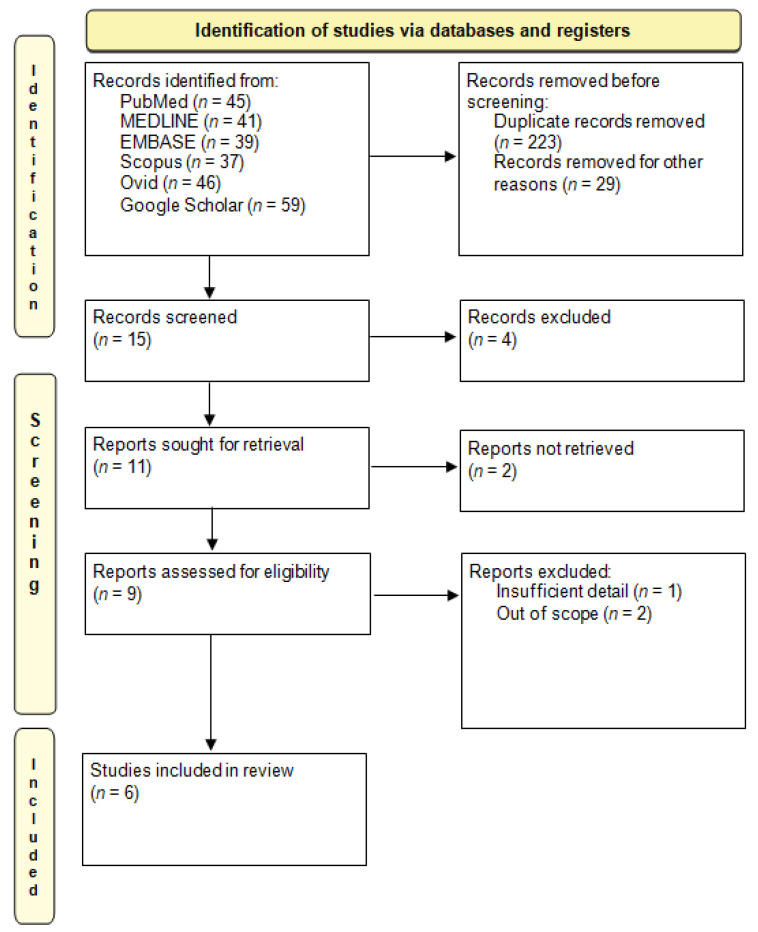
The Preferred Reporting Items for Systematic Reviews and Meta-analyses (PRISMA) 2020: flow chart illustrating the number of studies at each stage of the review.

The preliminary systematic search of the literature yielded 267 records. After excluding duplicates, 15 were considered potentially relevant and retained for the analysis of titles and abstracts. Thus, four were excluded as not meeting the characteristics of the approach or the review goal. After reviewing the full text of the remaining 11 records, among nine eligible studies, only six met the inclusion criteria and were included in the final qualitative analysis [12,16,21,22,23,24]. The PRISMA flow chart illustrating the number of studies at each stage of the review is shown in Figure 1.

The endpoint of the literary skimming process resulted in six eligible articles reported on a single exposure factor, TMD, and a single outcome, suicidal behavior, while assessed through different assessment tools [12,16,21,22,23,24]. Details of the design (cohort or cross-sectional), sample size (*n*) and sex ratio (%), minimum age and mean (SD), setting (community, hospital, home care), and country of individual studies are shown in Table 1.

Given the homogeneous shape of the recruitment setting for all selected studies (six out of the six), the distribution resulted as follows: 100% (*n* = 6) community. The Asian country led the geographical distribution of selected studies (66.6%, *n* = 4), followed by North America (16.7%, *n* = 1) and Europe (16.7%, *n* = 1). This latter perspective pointed to both the lack of homogeneity in geographical distribution and the total absence of representativeness of other countries. Mean (SD) age and sex ratio of study participants were recorded if applicable. In the totality of 44,645 subjects, females accounted for roughly the majority (about 65% vs. 35%). All selected studies reported a cross-sectional design (100%, *n* = 6).

### 3.1. Temporomandibular Disorders Assessment Tools

About the different types of TMD assessment tools, the World Health Organization (WHO) criteria (33.6%, *n* = 2) was the most frequently adopted, followed by the Research Diagnostic Criteria for Temporomandibular Disorders (RDC/TMD) (16.6%, *n* = 1), the Diagnostic Criteria for Temporomandibular Disorders (DC/TMD) (16.6%, *n* = 1), a self-reported experience of TM pain persisting 3 months or longer during the year (16.6%, *n* = 1), and finally, by an interview with accompanying oral clinical examination whose the criteria for diagnosis and classification of TMD are not reported (16.6%, *n* = 1).

### 3.2. Suicidal Behavior Assessment

In three of the six selected studies, suicidal behavior was assessed by asking patients a question that investigated the implementation of behaviors consistent with suicidal ideation, specifically: “Have you ever sincerely thought about committing suicide in the past year?” (33.6%, *n* = 2); “In the last 12 months, did you think about committing suicide” (16.6%, *n* = 1) [16,21,24]. Thoughts of suicide were assessed in a study [12] through the EuroQol-5 Dimension (EQ-5D) tool (16.6%, *n* = 1).

Suicidal ideation was assessed by item 9 on the PHQ-9 in another study [23] (thoughts that you would be better off dead or of hurting yourself in some way on more than half the days in the past two weeks), (16.6%, *n* = 1). Finally, in one study, three items of a validated questionnaire for psychopathological evaluation were used for the assessment of suicidal behavior, i.e., the full version of the Symptom Checklist 90 (SCL-90-R) (16.6%, *n* = 1) [22].

### 3.3. Summary of Findings on the Association of Temporomandibular Disorders and Suicidal Behavior

Examining the observational studies selected for this systematic review, we found a low association of TMD with suicidal behavior, with estimates partly provided [prevalence ratio (PR) from 1.26 to 1.35, 95% confidence intervals (CI) from 1.15 to 1.19 (lower) and from 1.37 to 1.54 (higher); and odds ratios (OR) from 1.54 to 2.56, 95% CI from 1.014 to 1.157 (lower) and 2.051 to 6.484 (higher)]. In particular, study-specific findings of the included studies suggested that suicidal behavior was more present in young adults with TMD (six of six studies), with controversial association with gender (three of six studies), and that suicidal behavior was correlated and aggravated by the intensity of pain (two of six studies). Finally, there was a worsening of symptoms such as anxiety and depression that may reduce the ability to adapt of patients with TDM (two of six studies) (Table 1 and Table 2).

### 3.4. Methodological Quality Assessment within Studies and Overall Quality Assessment across Studies

**Figure 2:**
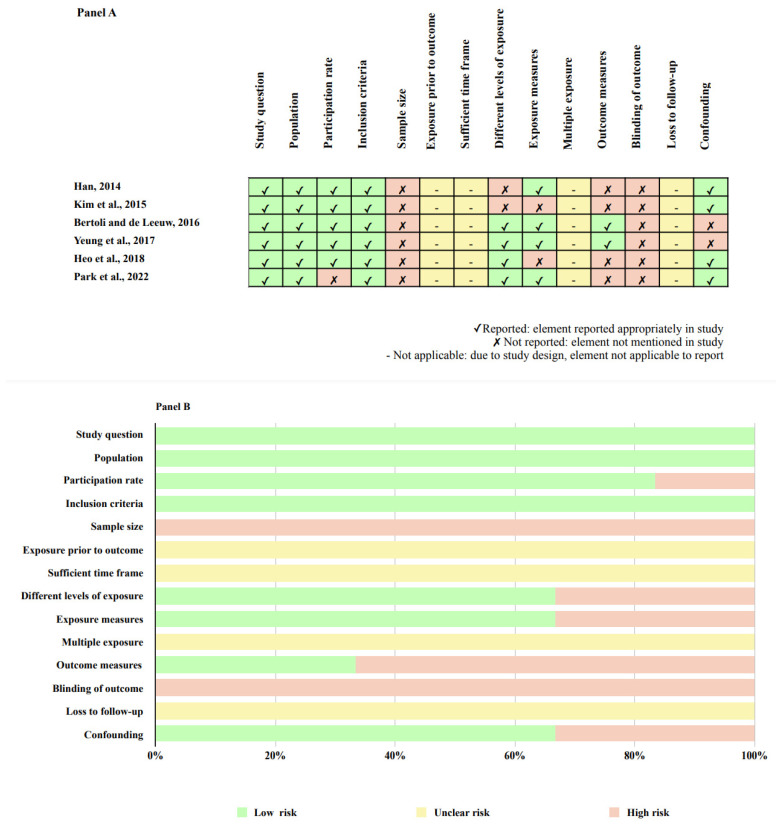
Methodological quality assessment within studies (**Panel A**) [12,16,21,22,23,24]; and overall quality assessment across studies (**Panel B**).

Examining all the six included studies, we found a low (*n* = 1), to moderate (*n* = 3) and high (*n* = 2) methodological quality (Table 1). An overview of quality ratings within (Panel A) and across studies (Panel B) was provided in Figure 2, highlighting areas with higher or lower risk ratings.

Bias was detected predominantly in the domains of sample size justification (selection bias), six of six studies (100%) were associated with a high risk of bias, and for blinded assessment (detection bias), six of six studies (100%) were associated with a high risk of bias. One (16.6%) study was associated with a higher risk of bias regarding the participation rate and also two out of six (33.3%) were found associated with a prevalent risk of bias in confounding (Figure 2, Panel B). The GRADE overall certainty of evidence was judged low for the assessed outcome (suicidal behavior) (Table 2).

## 4. Discussion

In the present study, we performed a systematic review of observational studies in which aspects related to TMD and suicidal behavior were highlighted, the outcomes sought mainly concerned aspects related to prevalence data and the criteria used to identify suicidal behavior. In the present systematic review, we found a low association of TMD with suicidal behavior, with estimates partly provided [PR from 1.26 to 1.35, 95% CI from 1.15 to 1.19 (lower) and from 1.37 to 1.54 (higher); and OR from 1.54 to 2.56, 95% CI from 1.014 to 1.157 (lower) and 2.051 to 6.484 (higher)], a relevant sample size of 44,645 patients, but only 6 studies included. In particular, study-specific findings of the included reports showed that suicidal behavior was more present in young adults with TMD, with controversial association with gender. Suicidal behavior was also correlated and aggravated by the intensity of pain. Finally, there was a worsening of anxiety and depression symptoms that may reduce the ability to adapt of TDM patients, and this condition could lead to suicidal behavior.

To the best of our knowledge, the present study is the first systematic review investigating possible associations between TMD and suicidal behaviour. Among the selected studies, Bertolli and de Leeuw in 2016 split the cohort of their retrospective study in muscle pain patients and joint pain patients [22]. They reported that pain severity was higher in the muscle pain group, with a higher prevalence in the latter group of thoughts of ending their life (11.5%). In agreement with previous studies, they also reported anxiety and depression data with a prevalence of 30.4% and 28.9%, respectively [22].

In 2014, Han identified TMD through three signs reported by patients participating in the KNHANES IV study [21]: the appearance of mandibular clicks upon opening, tenderness (on palpation), and reduced mobility of the jaw. The dichotomous question for the identification of suicidal thoughts (yes or no) was the following: “In the last 12 months, have you thought about killing yourself? Contrary to previous studies, Heo and colleagues, examining KNHANES IV data, considered an age range between 12 and 18 years. They reported that the male sex was more associated to TMD; also, suicidal ideation was more present in the male sex [24]. The authors justified this apparent disagreement due to a Korean society which generates a greater expectation in male adolescents [25].

Park and colleagues, in a study conducted using again KNHANES IV data, considered all the age groups [16]. These findings provided evidence that TMD was associated with stress in both genders and was associated with suicidal ideation, especially in women. But the association with gender is controversial. TMD is known to affect quality of life, large-scale studies investigating the influence of this disorder on quality of life by gender were scarce. Kim and colleagues suggested that while TMD itself was negatively associated with general quality of life, comorbidities such as osteoarthritis and mental health issues may strengthen this negative relationship [12]. The association was stronger in women, which may reflect susceptibility to chronic pain and mental health disorders [12]. In 2007, Yeung and colleagues highlighted the importance of screening with appropriate and integrated tools to identify suicidal thoughts in the population with both TMD and chronic pain [23]. Furthermore, the present data showed that patients in whom the treatment of TMDs was less effective (16% in Bertoli and de Leeuw [22]) reported greater psychological symptoms with inability to implement adaptive behaviors, in addition to negative expectations about their own ability to manage pain, and this condition could lead to suicidal ideation [26].

**Figure 3:**
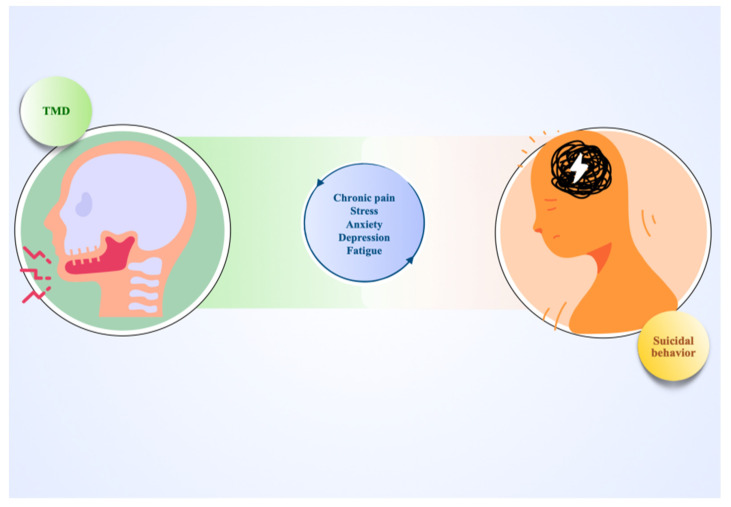
Schematic representation of some possible underlying mechanisms explaining the association between the presence of temporomandibular disorders (TMD) and suicidal behaviour.

In Figure 3, some possible underlying mechanisms explaining the association between the presence of TMD and suicidal behaviour. Suicidal ideation represents only one aspect of the set of symptoms related to suicide, but it is important to understand both the onset of subjects with chronic pain attributable to TMD and its significance as a risk factor for self-destructive behaviors, because it itself is a mirror of emotional suffering [27]. The OPPERA team has identified and explored clinical, psychological, and sensory phenotypes of TMD [28].

Furthermore, persons with TMD had increased levels of proinflammatory cytokines, which correlate with greater pain sensitivity, perceived stress, and depression [29]. The pain, joint noise, and jaw movement dysfunction in TMD negatively impinge the lifestyles of those affected. Inflammatory cytokines secreted into synovial fluid of TMD in inflamed sites may be involved in augmentation of inflammation, transition to chronicity, and the development of psychiatric comorbidities [30]. Furthermore, suicidal behaviour could be associated to an inflammatory pattern [31,32]. Therefore, a goal in personalized medicine, would be to early identify and recognize suicidal behavior in subjects with TMD/chronic pain, taking into account possible completion of suicide, in order to frame the patient who would be directed towards a treatment that is not only of dental relevance.

Some limitations of the present study must be acknowledged. The main limitation of this systematic review was identified in a lack of clinical studies in the two areas (TMD and suicidal behavior). In fact, we found only six reports to be included. Furthermore, the majority of the studies (four out of six) were performed in Korea, so there could be a bias related to territoriality. However, Korea is one of the countries with the main prevalence of suicidal behavior, with a great interest in this topic [33]. In addition, the assessment of the included studies reported data with a low certainty of evidence.

## 5. Conclusions

In conclusion, examining the findings of the selected studies, suicidal behavior was more present in young adults with TMD, with controversial association with gender. Furthermore, suicidal behavior was correlated and aggravated by the intensity of pain. Finally, there was a worsening of symptoms such as anxiety and depression that may reduce the ability to adapt of patients with TDM and this condition could lead to suicidal behavior. In the near future, to increase the low strength of evidence of the collected findings and to address the regional bias of the included reports, larger studies, with a population-based design, longer follow-up periods, conducted in different countries/geographical areas and evaluating the association between TMD and incident suicidal behavior are needed, addressing also potential bias and confounding sources.

## Figures and Tables

**Table 1:** Selected studies investigating temporomandibular disorders and suicidal behavior (*n* = 6) and quality appraisal summary. M: males; F: females; TMD: temporomandibular disorders.

Authors, Year (Reference)	TMDs Assessment Tool(s)	Outcome(s)’ Assessment	Design	*n*	Age	Setting(s)	Country	Quality Assessment	Main Findings
Han, 2014 [21]	World Health Organization (WHO) criteria	“In the last 12 months, did you think about committing suicide”	Cross-sectional	16658 (42.3% M, 57.7% F)	>19 years old	Community	Asia (South Korea)	Moderate	Pain conditions, including TMD pain, may aggravate suicidal ideation among those with a cancer history
Kim et al., 2015 [12]	Self-reported (experience of TM pain persisting 3 months or longer during the year)	EuroQol-5 Dimension	Cross-sectional	17198 (42.4% M, 57.6% F)	>19 years old	Community	Asia (South Korea)	Moderate	Chronic diseases and psychological factors are important in chronic TMD, and there may be physiological and pathological gender differences in TMD
Bertoli and de Leuwee, 2016 [22]	Research Diagnostic Criteria for TMD (RDC/TMD)	3 items of the Symptom Checklist 90 (SCL-90R) (item 15): “thoughts of ending your life (item 54): “feeling hopeless about the future” (item 59): “thoughts of death and dying”	Cross-sectional	1241 (11.7% M, 88.3% F)	>18 years old; mean age 35.76 ± 12.6	Community	North America (USA)	High	Elevated levels of suicidal ideation, depression and anxiety were reported in a chronic TMD population, especially in those with chronic muscle pain, compared to the general population
Yeung et al., 2017 [23]	Diagnostic Criteria for TMD (DC/TMD)	Item 9 of the Patient Health Questionnaire-9 (PHQ-9)	Cross-sectional	162 (20% M, 80% F)	Median age 35 years old (27–47)	Community	Europe (United Kingdom)	High	Early identification of mental health problems and concurrent management may be most beneficial in subjects with TMD symptoms for up to two years
Heo et al., 2018 [24]	Interviews and oral examination	“Have you ever sincerely thought about committing suicide in the past year?”	Cross-sectional	1337 (53.6% M; 46.4% F)	12–18 years old	Community	Asia (South Korea)	Low	The results provide evidence that the depressed mood and suicidal ideation were associated with TMD in male adolescents
Park et al., 2022 [16]	World Health Organization (WHO) criteria	“Have you ever sincerely thought about committing suicide in the past year?”	Cross-sectional	8049 (42.2% M, 57.8% F)	19–59 years old	Community	Asia (South Korea)	Moderate	This study found that suicidal ideation is closely associated with TMD in women

**Table 2:** GRADE summary of findings on temporomandibular disorders (TMD) associated to suicidal behavior.

Outcome	Number of Participants (Studies) Design	Study-Specific Results and Measurements	Certainty of Evidence (GRADE)
Suicidal behavior	44,645 patients (six studies) [12,16,21,22,23,24] All cross-sectional	Total TMD vs. suicidal ideation: PR: 1.26, 95% CI: 1.15–1.37 (*p* < 0.001); Severe TMD vs. suicidal ideation: PR: 1.35, 95% CI: 1.19–1.54 (*p* < 0.001) [21] Chronic TMD vs. thoughts of suicide: ß = -0.028 (*p* < 0.0001) [12] Pain severity vs. SCL-90-R (item 15): ρ = 0.099 (*p* < 0.001); Pain severity vs. SCL-90-R (item 54): ρ = 0.137 (*p* < 0.001); Pain severity vs. SCL-90-R (item 59): ρ = 0.109 (*p* < 0.001) [22] TMD vs. Patient Health Questionnaire-9 (PHQ–9) = 3.6 (*p* = 0.033) [23] TMD (males) vs. suicidal ideation: OR: 2.56, 95% CI: 1.014–6.484 (*p* < 0.05) [24] TMD (women) vs. suicidal ideation: OR: 1.54, 95% CI: 1.157–2.051 (*p* < 0.05) [16]	⊕⊕ Low ^a^

TMD: temporomandibular disorders; PR: prevalence ratio; CI: confidence interval; SCL-90-R: Symptom Checklist 90; OR: odds ratio. **^a^ Suicidal behaviour: Risk of bias: Serious**. Lack of blinding of outcome assessors, resulting in potential for detection bias. Lack of sample size justification, resulting in potential for selection bias; decided not to rate down further for risk of bias as we included only obervational studies; **Inconsistency: Not serious**; **Imprecision: Not serious**. Not wide confidence intervals; **Publication bias: Not serious**. Less that 10 studies. ⊕⊕: Low (according to GRADE Certainty of Evidence).

## Data Availability

Not applicable.

## Supplementary Materials

The following supporting information can be downloaded at: https://www.mdpi.com/article/10.3390/jpm12111782/s1, Table S1: Search strategy used in the US National Library of Medicine (PubMed) and Medical Literature Analysis and Retrieval System Online (MEDLINE) and adapted to the other sources, according to selected descriptors.
