# Supplementary material for: Temporomandibular Disorders as a Risk Factor for Suicidal Behavior: A Systematic Review
Source: J Pers Med. 2022 Oct 28;12(11):1782. doi: 10.3390/jpm12111782 (PMC9692338; doi:10.3390/jpm12111782)
Supplement: Supplementary file 1 [file jpm-12-01782-s001.zip › jpm-1954341-supplementary.pdf]

Table S1. Search strategy used in the US National Library of Medicine (PubMed) and Medical Literature Analysis and Retrieval System Online (MEDLINE) and adapted to the other sources, according to selected descriptors.

**Strategy**	**Descriptors used**
# 1	(suicidal ideation[tiab]) OR (suicide[tiab]) OR (suicide, attempted[tiab]) OR (suicidality[tiab]) OR (parasuicide[tiab]) OR (parasuicidal[tiab]) OR (completed suicide[tiab]) OR (suicidal behavior[tiab]) OR (self-harm[tiab]) OR (self-directed violence[tiab])
# 2	(temporomandibular disorders[tiab]) OR (TMD[tiab]) OR (craniomandibular disorders[tiab]) OR (cranio-mandibular disorders[tiab]) OR (temporo-mandibular disorders[tiab]) OR (TMJ[tiab]) OR (TMJD[tiab]) OR (Costen syndrome[tiab])
# 3	(review[tiab]) OR (narrative review[tiab]) OR (systematic review[tiab]) OR (editorial[tiab]) OR (perspective[tiab]) OR (letter[tiab]) OR (comentary[tiab])
# 5	#1 AND #2 NOT #3
Sort by: Most Recent. Number of papers: 45 Date: 25 March 2022	
